# Risk factors for hospitalization and pneumonia development of pediatric patients with seasonal influenza during February–April 2023

**DOI:** 10.3389/fpubh.2023.1300228

**Published:** 2024-01-05

**Authors:** Yuqian Zhang, Xing Huang, Jianguo Zhang, Zhimin Tao

**Affiliations:** ^1^Department of Emergency Medicine, The Affiliated Hospital, Jiangsu University, Zhenjiang, Jiangsu, China; ^2^Center for Evidence-Based and Translational Medicine, Zhongnan Hospital of Wuhan University, Wuhan, China; ^3^Jiangsu Province Key Laboratory of Medical Science and Laboratory Medicine, Department of Laboratory Medicine, School of Medicine, Jiangsu University, Zhenjiang, Jiangsu, China

**Keywords:** influenza infection, risk factor, pediatric hospitalization, pneumonia, post-COVID

## Abstract

**Objectives:**

In China influenza remains a low activity for continuous 3 years due to COVID-19 controls. We here sought to study the clinical characteristics and risk factors of the influenza infection among children after the mandatory COVID-19 restrictions were lifted.

**Methods:**

We included 1,006 pediatric patients with influenza A virus (IAV) infection, enrolled in one tertiary hospital in Zhenjiang, Jiangsu Province, China, during February to April 2023. Patients were divided into the outpatient (*n* = 798) and inpatient (*n* = 208) groups, and their baseline characteristics were compared between two groups to conclude the risk factors for pediatric hospitalization. Separately, pediatric inpatients (*n* = 208) were further divided into the pneumonia and non-pneumonia groups with comparison of their clinical characteristics, including their laboratory test results and representative radiological features, to derive the key determinants for pneumonia development after hospitalization.

**Results:**

Compared to outpatients, IAV-infected pediatric inpatients exhibited younger age, higher female: male ratio, more co-infection of influenza B virus (IBV) and hematological abnormality. Multivariate regression analysis determined the independent risk factors of hospitalization to be the clinical symptom of abdominal pain (OR = 2.63, [95% CI, 1.05–6.57], *p* = 0.039), co-infection of IBV (OR = 44.33, [95% CI, 25.10–78.30], *p* = 0.001), elevated levels of lymphocytes (OR = 2.24, [95% CI,1.65–3.05], *p* = 0.001) and c-reactive proteins (CRPs) (OR = 1.06, [95% CI, 1.03–1.08], *p* = 0.001) upon hospital admission. Furthermore, the cough symptom (OR = 17.39, [95% CI, 3.51–86.13], *p* = 0.001) and hospitalization length (OR = 1.36, [95% CI, 1.12–1.67], *p* = 0.002) were determined to be risk factors of pneumonia acquirement for pediatric inpatients.

**Conclusion:**

While the abdominal pain, viral co-infection and some hematological abnormality mainly contribute to hospitalization of pediatric patients with IAV infection, the length of hospital stay and clinical sign of coughing upon hospital admission constitute the key determinants for nosocomial pneumonia development.

## Introduction

In a non-pandemic year, 1 billion cases of seasonal influenza are estimated worldwide, of which 3–5 million infections might result in severe illness, causing 290,000–650,000 influenza-associated fatality (i.e., 0.1–0.2% mortality rate) (1). The infection rate among the unvaccinated individuals is 22.5% for children (<18 years) and 10.7% for adults, albeit nearly half of infections are asymptomatic (2). Age, obesity, chronic comorbidity and weakened immunity reportedly constitute the elevated risks for influenza complications (3). The United States (U.S.) population-based surveillance network reported that the hospitalization rate of patients with influenza during 2021–2022 season was 17.3 per 100,000 persons, with a high rate of 30.9% among the children (<18 years) and 31 influenza-associated pediatric deaths (4). Influenza has been a considerable cause of pediatric visit and hospitalization due to the lack of immunity and the increase of virulence, although the exact risk factors for flu complications in children remain unclear (5).

In the past two winters, due to mandatory COVID-19 measures (e.g., mask wearing, social distancing and travel restricting), influenza incidence was unprecedentedly low and influenza-caused hospitalization dropped to the lowest level in decades (6). However, as China’s national policy to prevent and control COVID-19 was shifted on December 7, 2022, the positive rate of SARS-CoV-2 in population continued to rise and peaked on December 23–25 (7). In parallel, the positive rate of influenza virus remained constantly low until early February 2023, but began to climb and reached the highest in late March before it gradually declined (8). This flu eruption was predicted due to the loosening of COVID-19 restrictions, as well as the loss of natural immunity developed for the circulating influenza virus in the past seasons prior to the year of 2023 (9).

In this study we reported 1,006 pediatric patients with laboratory-confirmed influenza illness from The Affiliated Hospital of Jiangsu University (TAHJU), Jiangsu Province, China, during February-April 2023, a period when relatively low SARS-CoV-2 and high influenza virus activities co-existed in the region (10, 11). We analyzed and compared the clinical characteristics between pediatric patients in the outpatient and inpatient units, with an aim to determine the risk factors of hospitalization due to seasonal influenza infection in children. Furthermore, we investigated the clinical manifestation of hospitalized pediatric patients with seasonal influenza circulating in the early year 2023, revealing the key determinants for pneumonia development.

## Methods

### Study design and patient information

**Figure 1:**
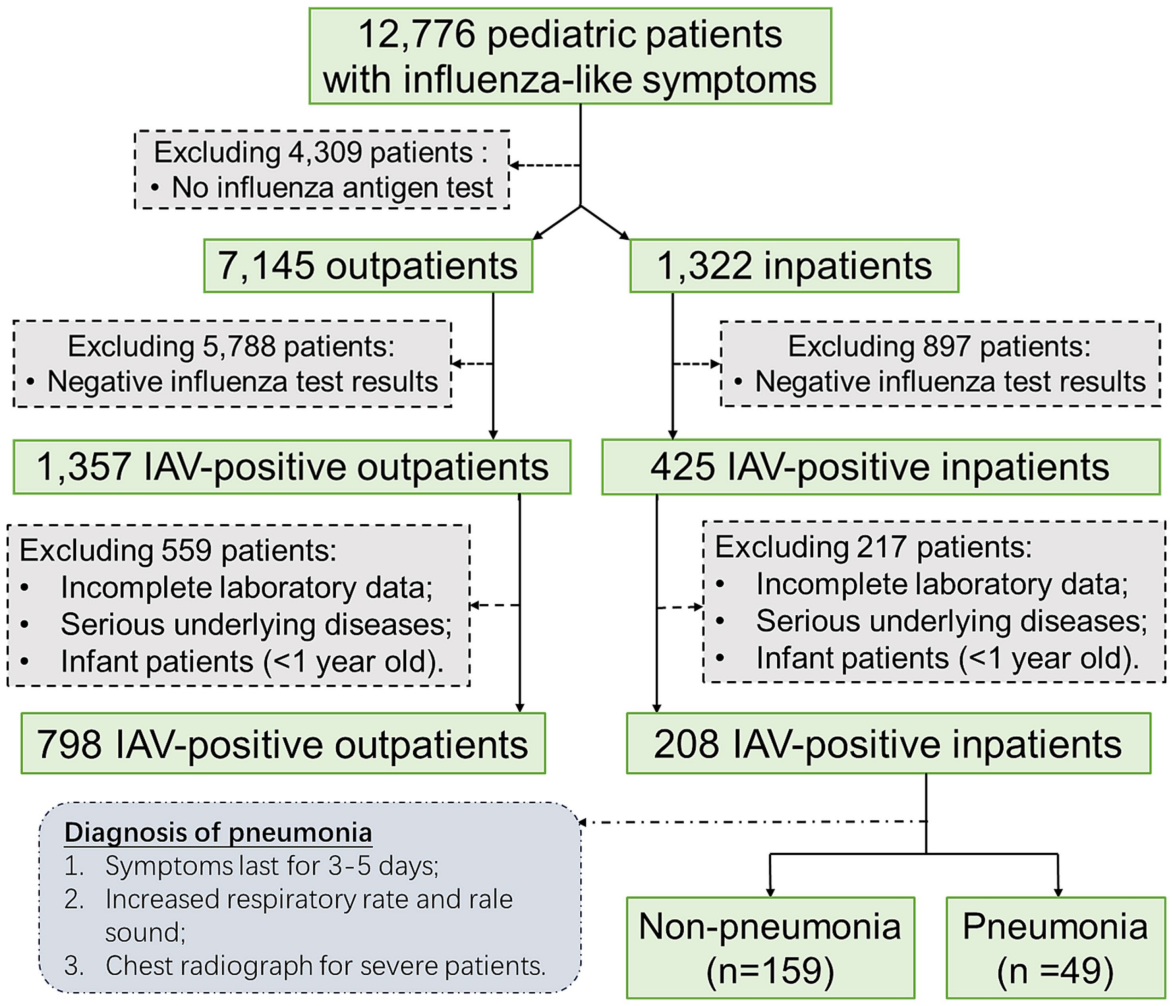
The flowchart illustrates the inclusion and exclusion criteria of pediatric patients in the study.

This retrospective study included 1,006 pediatric patients who were infected with influenza A virus (IAV) and admitted to The Affiliated Hospital of Jiangsu University (TAHJU), Zhenjiang City, Jiangsu Province, China, from February to April 2023. The inclusion and exclusion criteria of pediatric patients were as shown in Figure 1, where patients with certain underlying diseases that may seriously impact their blood profiles, including malignancy, hematological disorders, or autoimmune deficiency were excluded. The study was approved by the Research Ethics Committee of TAHJU, and the patient consents were acquired from their parents or legal guardians.

### Procedure

The diagnosis and treatment of influenza were following the reported guideline (12), and pediatric patients with 1 < age < 18 years were included in this study. Briefly, following the virus isolation and culture, identification of influenza virus antigen and nucleic acids can be used for early diagnosis, and detection of virus-specific IgG antibodies can be useful during the acute or recovery phase. The diagnosis of mycoplasma infection followed *Guidelines for the Diagnosis and Treatment of Mycoplasma Pneumoniae in Children (2023)* (in Chinese), which was summarized below: (1) clinical presentation of fever and cough; (2) chest imaging with infiltrates; (3) positive detection of *Mycoplasma Pneumoniae* DNA or RNA; (4) *Mycoplasma Pneumoniae* serum antibody titer ≥1: 160 or four-fold rising titer in acute and convalescent serum specimens (13). For hospitalized children, pneumonia development was diagnosed by following the *Guideline for Diagnosis and Treatment of Community-acquired Pneumonia in Children (in Chinese)* (14). In brief, the patients’ symptoms were first assessed, especially for those with persistent fever and cough for more than 3 days. Increased respiratory rates and presence of rales are indicative of pneumonia. Chest radiographs might not be performed on patients with mild degree of illness, and usually computed tomography (CT) is not recommended. Pediatric patients with pneumonia can be confirmed with chest radiographs showing patchy shadowing, typically centered in the middle and lower lobes of the lungs (15). Serological tests were based on immunochromatographic assay, using a nine-item IgM antibody test kit for respiratory tract infection pathogens (VIRCELL, S.L., China), to simultaneously detect nine microorganisms that include *Mycoplasma Pneumoniae*, adenovirus, respiratory syncytial virus, IAV, IBV and parainfluenza, etc. Blood cell analysis was conducted by an automated XN1000 hematology (SYSMEX, Japan), and biochemical indicators were analyzed using VITROS 350 autoanalyzer (Johnson & Johnson, USA).

### Statistical analysis

Data were summarized as the median and interquartile range (IQR) values for continuous variables and frequencies for categorical variables. For comparisons between the two groups, the Mann–Whitney U test was used for continuous variables. Categorical variables were examined by *χ*^2^ test. All calculated *p* values were two-sided, and *p* < 0.05 was considered statistically significant. Both data were subjected to binary logistic regression. All statistical analyses were performed using SPSS version 16.0.

## Results

### Risk factors associated with hospitalization of pediatric patients with IAV infection

This retrospective study included 1,006 patients infected with IAV that were further divided into two groups: i.e., 798 (79.3%) outpatients and 208 (20.7%) inpatients. Their baseline characteristics, including demographic information, clinical signs, and blood cell counts, were analyzed, and compared between two groups (Table 1). For all patients, the median age was 6.0 (IQR: 4.0–9.0), and 45.9% were female. 29.9% of patients were co-infected with IBV. None of pediatric patients experienced asymptomatic influenza. Fever topped in the list of common clinical symptoms, followed by cough, vomiting, muscle pain, sore throat, expectoration, and abdominal pain. Compared to the outpatients, the inpatients showed significantly younger age, higher female proportion, and much more IBV co-infection. For clinical signs, the inpatients had a higher frequency of cough, vomiting, expectoration, and abdominal pain, but a lower incidence of fever, muscle pain and sore throat.

**Table 1:** Baseline characteristics of pediatric patients with IAV infection.

	Total (*n* = 1,006)	Outpatient (*n* = 798)	Inpatient (*n* = 208)	*p*
Age (year)	6.0 (4.0–9.0)	7.0 (5.0–9.0)	5.0 (4.0–7.0)	0.001
Gender, female (%)	462 (45.9%)	351 (44.0%)	111 (53.4%)	0.016
IVB co-infection	301 (29.9%)	120 (15.0%)	181 (87.0%)	0.001
Symptoms				
Fever	990 (98.4%)	792 (99.2%)	198 (95.2%)	0.001
Cough	519 (51.6%)	396 (49.6%)	123 (59.1%)	0.015
Vomiting	155 (15.4%)	109 (13.7%)	46 (22.1%)	0.003
Muscle pain	154 (15.3%)	137 (17.2%)	17 (8.2%)	0.001
Sore throat	117 (11.6%)	104 (13.0%)	13 (6.3%)	0.007
Expectoration	86 (8.5%)	61 (7.6%)	25 (12.0%)	0.044
Abdominal pain	72 (7.2%)	49 (6.1%)	23 (11.1%)	0.014

For all patients with influenza, a group of patients presented blood parameters that fell out of the normal range. Based on the lymphocyte counting, it did show the statistical significance in the difference between pediatric inpatients and outpatients; however, the abnormality of increased lymphocytes was not prevalent in outpatients, although the inpatients showed a noticeable portion (9.1%) with abnormally high counts of lymphocytes. Similarly, although the count of red blood cells (RBCs) did not exhibit much abnormality in both groups, the level of hemoglobin and the values of hematocrit, MCV, and MCH were significantly lower in the inpatient group, indicating their worse anemic condition or association with the younger age of the inpatients. Differently, a substantial portion of both inpatients and outpatients showed deranged CRP values, while the CRP level of inpatients was significantly higher than that of outpatients, pointing out more severe inflammation.

In order to verify whether there are independent risk factors influencing hospitalization of pediatric patients, we adopted the multivariable regression analysis by inputting all parameters with significant difference between two groups. Results are shown in Table 2. It depicts that in our study age and gender are not independent risk factors associated with pediatric hospitalization. Upon hospital visit, the fever symptom, the monocytosis, and the low hemoglobin level could render the children low odds for further hospitalization. Conversely, the sign of abdominal pain, the IBV co-infection, and the levels of lymphocytes and CRP, put the pediatric patients infected with IAV higher odds for hospitalization (Table 3).

**Table 2:** Basic hematological profile of pediatric patients with IAV infection upon their hospital visit.

	Normal range	Total (*n* = 1,006)	Outpatient (*n* = 798)	Inpatient (*n* = 208)	*p*
White blood cells, ×10^9^/L	3.5–9.5	5.4 (4.2–6.9)	5.4 (4.3–6.8)	5.4 (3.9–8.2)	0.962
>9.5		63 (6.3%)	32 (4.0%)	31 (14.9%)	0.001
Neutrophils, ×10^9^/L	1.8–6.3	3.6 (2.5–4.9)	3.6 (2.6–4.9)	3.4 (1.8–5.3)	0.138
>6.3		113 (11.2%)	76 (9.5%)	37 (17.8%)	0.001
Lymphocytes, ×10^9^/L	1.1–3.2	1.0 (0.7–1.5)	1.0 (0.7–1.4)	1.2 (0.7–2.3)	0.001
>3.2		28 (2.8%)	9 (1.1%)	19 (9.1%)	0.001
Monocytes, ×10^9^/L	0.1–0.6	0.5 (0.4–0.7)	0.6 (0.4–0.7)	0.5 (0.3–0.6)	0.001
>0.6		307 (30.5%)	259 (32.5%)	48 (23.1%)	0.009
Red blood cells, ×10^12^/L	3.8–5.1	4.6 (4.4–4.8)	4.7 (4.5–4.9)	4.3 (4.1–4.6)	0.001
<3.8		13 (1.3%)	7 (0.9%)	6 (2.9%)	0.053
Hemoglobin, g/L	115.0–150.0	130.0 (123.0–137.0)	132.0 (126.0–139.0)	121.0 (115.0–127.0)	0.001
<115.0		78 (7.8%)	28 (3.5%)	50 (24.0%)	0.001
Hematocrit, %	35.0–50.0	38.9 (36.8–41.0)	39.5 (38.1–41.6)	35.6 (34.2–37.3)	0.001
<35.0		97 (9.6%)	26 (3.3%)	71 (34.1%)	0.001
MCV, fL	82.0–100.0	84.7 (82.2–87.2)	85.3 (82.9–87.8)	82.5 (80.0–84.8)	0.001
<82.0		229 (22.8%)	136 (17.0%)	93 (44.7%)	0.001
MCH, pg	27.0–34.0	28.2 (27.4–29.1)	28.3 (27.6–29.1)	28.0 (27.0–28.7)	0.001
<27.0		168 (16.7%)	117 (14.7%)	51 (24.5%)	0.001
MCHC, g/L	316.0–354.0	333.0 (327.0–340.0)	332.0 (326.0–338.0)	338.0 (332.0–343.0)	0.001
<316.0		26 (2.6%)	25 (3.1%)	1 (0.5%)	0.032
RDW, %	11.0–16.0	12.8 (12.4–13.3)	12.8 (12.5–13.3)	12.6 (12.2–13.0)	0.001
>16.0		2 (0.2%)	2 (0.3%)	0 (0%)	1.000
Platelet, ×10^9^/L	125.0–350.0	210.0 (177.0–246.0)	210.0 (179.0–245.0)	208.0 (165.0–258.0)	0.945
<125.0		23 (2.3%)	11 (1.4%)	12 (5.8%)	0.001
MPV, fL	7.4–12.5	10.0 (9.3–10.8)	10.1 (9.5–10.8)	9.6 (8.9–10.5)	0.001
>12.5		26 (2.6%)	23 (2.9%)	3 (1.4%)	0.244
PDW, %	9.0–17.0	12.5 (10.7–15.8)	12.1 (10.6–15.7)	15.6 (12.2–15.9)	0.001
>17.0		23 (2.3%)	22 (2.8%)	1 (0.4%)	0.090
CRP, mg/L	0–10.0	2.8 (0.5–6.8)	2.4 (0.5–6.4)	3.8 (1.2–10.7)	0.001
>10.0		162 (16.1%)	99 (12.4%)	63 (30.3%)	0.001

MCV, mean corpuscular volume; MCH, mean corpuscular hemoglobin; MCHC, mean corpuscular hemoglobin concentration; RDW, red blood cell distribution width; MPV, mean platelet volume; PDW, platelet distribution width; CRP, c-reactive protein.

**Table 3:** Multivariable regression analysis of independent risk factors for hospitalization of pediatric patients.

	Odds ratio (OR)	95% Confidence interval (CI)	*p*
Basic feature			
Age (year)	1.31	0.84–1.03	0.159
Gender, female (%)	0.93	0.81–2.11	0.270
Co-infection			
IBV	44.33	25.10–78.30	0.001
Symptoms			
Fever	0.05	0.01–0.32	0.002
Cough	1.56	0.94–2.58	0.083
Expectoration	1.06	0.45–2.49	0.893
Abdominal pain	2.63	1.05–6.57	0.039
Vomiting	1.46	0.78–2.74	0.234
Blood cell count			
Lymphocytes, ×10^9^/L	2.24	1.65–3.05	0.001
Monocytes, ×10^9^/L	0.17	0.06–0.50	0.001
Hemoglobin, g/L	0.91	0.89–0.94	0.001
CRP, mg/L	1.06	1.03–1.08	0.001

### Clinical characteristics of pediatric inpatients and risk factors for pneumonia development

For 208 hospitalized pediatric patients, we next investigated their main clinical manifestations and assessed the key determinants associated with further pneumonia development. During hospitalization, 49 (23.6%) pediatric patients developed pneumonia. As shown in Table 4, the median age of inpatients was 5.0 (IQR: 4.0–7.0) years, and their median length of hospitalization was 5.0 (IQR: 4.0–6.0) days, where the pneumonia group had significantly longer hospital stay than the non-pneumonia group. Among inpatients, 53.4% were female, and body mass index (BMI) showed no difference between two groups. Compared to the non-pneumonia group, the pneumonia group presented higher frequency of clinical symptoms that included cough and expectoration, but less frequency of convulsion. While the most inpatients were confirmed with co-infections, the pneumonia group had significantly higher incidence of mycoplasma infection than the non-pneumonia group. Concurrently, the increased number of co-infections was not linked to increased occurrence in patients that acquired pneumonia.

**Table 4:** Baseline characteristics of pediatric inpatients with IAV infection.

	Total (*n* = 208)	Non-pneumonia (*n* = 159)	Pneumonia (*n* = 49)	*p*
Age (year)	5.0 (4.0–7.0)	5.0 (4.0–7.0)	6.0 (4.0–7.0)	0.870
Length of stay (day)	5.0 (4.0–6.0)	5.0 (3.0–6.0)	7.0 (5.0–8.0)	0.001
Gender, female *N* (%)	111 (53.4%)	80 (50.3%)	31 (63.3%)	0.112
BMI (kg/m^2^)	16.5 (15.4–18.7)	16.6 (15.6–18.7)	16.3 (15.1–18.8)	0.222
Symptoms				
Fever	198 (95.2%)	149 (93.7%)	49 (100.0%)	0.072
Cough	123 (59.1%)	76 (47.8%)	47 (95.9%)	0.001
Vomiting	46 (22.1%)	39 (24.5%)	7 (14.3%)	0.131
Expectoration	25 (12.0%)	11 (6.9%)	14 (28.6%)	0.001
Abdominal pain	23 (11.1%)	20 (12.6%)	3 (6.1%)	0.208
Convulsion	19 (9.0%)	19 (11.9%)	0 (0%)	0.011
Muscle pain	17 (8.2%)	15 (9.4%)	2 (4.1%)	0.232
Sore throat	13 (6.3%)	12 (7.5%)	1 (2.0%)	0.164
Diarrhea	6 (2.9%)	6 (3.8%)	0 (0%)	0.168
Co-infection				
IBV	181 (87.0%)	137 (86.2%)	44 (89.8%)	0.508
Mycoplasma	42 (20.2%)	25 (15.7%)	17 (34.7%)	0.004
Parainfluenza virus	7 (3.4%)	4 (2.5)	3 (6.1)	0.441
EB virus	4 (1. 9%)	1 (0.6%)	3 (6.1%)	0.064
Tuberculosis	1 (0.5%)	1 (0.6%)	0 (0%)	1.000
Number of co-infections				
1	137 (65.9%)	111 (69.8%)	26 (53.1%)	0.031
2	42 (20.2%)	26 (16.4%)	16 (32.7%)	0.013
2+	3 (1.4%)	1 (0.6%)	2 (4.1%)	0.277

A substantial proportion of pediatric patients demonstrated abnormal cell counts, in which the pneumonia group presented worse degrees of leukocytosis, lymphocytosis, and monocytosis, although they showed comparable conditions to those in the non-pneumonia group where anemia and thrombocytopenia were uncommon (Table 5). Notably, several biochemical indicators that had a substantial proportion of patients with abnormal values, including procalcitonin, AST, ALP, sodium level, reflected more severe conditions in the non-pneumonia group than those in the pneumonia group. In addition, many parameters demonstrated significant differences between two groups, such as ALT, BUN, and albumin (data not shown); however, the proportion of patients showing abnormality of those blood parameters was very marginal and so negligible when considering whether they were factorial in pneumonia acquirement.

**Table 5:** Hematological profile of pediatric inpatients with IAV infection upon their hospital admission.

	Normal range	Total (*n* = 208)	Non-pneumonia (*n* = 159)	Pneumonia (*n* = 49)	*p*
Blood cell count					
White blood cells, ×10^9^/L	3.5–9.5	5.4 (3.9–8.2)	5.1 (3.6–7.1)	6.0 (4.5–8.6)	0.027
>9.5		31 (14.9%)	22 (13.8%)	9 (18.4%)	0.436
Lymphocytes, ×10^9^/L	1.1–3.2	1.2 (0.7–2.3)	1.1 (0.6–1.8)	2.1 (1.1–3.2)	0.001
>3.2		19 (9.1%)	10 (6.3%)	9 (18.4%)	0.022
Monocytes, ×10^9^/L	0.1–0.6	0.5 (0.3–0.6)	0.4 (0.3–0.6)	0.5 (0.4–0.7)	0.008
>0.6		48 (23.1%)	32 (20.1%)	16 (32.7%)	0.069
Platelet, ×10^9^/L	125.0–350.0	208.0 (165.0–258.0)	200.0 (161.0–250.0)	232.0 (189.0–279.0)	0.009
<125.0		12 (5.8%)	10 (6.3%)	2 (4.1%)	0.819
Biochemical panel					
Procalcitonin, ng/mL	<0.1	0.1 (0.1–0.3)	0.2 (0.1–0.4)	0.1 (0.1–0.2)	0.003
>0.1		116 (55.8%)	96 (60.4%)	20 (40.8%)	0.016
ALT, U/L	9.0–50.0	20.0 (16.0–24.0)	20.0 (17.0–25.0)	18.0 (16.0–22.5)	0.030
>50.0		10 (4.8%)	7 (4.4%)	3 (6.1%)	0.912
AST, U/L	15.0–40.0	49.0 (39.0–68.0)	51.0 (41.0–71.0)	43.0 (35.5–60.0)	0.024
>40.0		149 (71.6%)	119 (74.8%)	30 (61.2%)	0.064
ALP, U/L	32.0–126.0	159.0 (130.0–197.0)	170.0 (135.0–204.0)	150.0 (122.5–162.0)	0.001
>126.0		161 (77.4%)	125 (78.6%)	36 (73.5%)	0.451
Sodium, mmol/L	137.0–147.0	135.6 (133.3–137.3)	135.0 (133.0–137.0)	136.9 (134.9–138.7)	0.001
<137.0		145 (69.7%)	118 (74.2%)	27 (55.1%)	0.011

Only data with significant difference (*p* < 0.05) between two groups and having a noticeable portion of patients with abnormality (>5%) were shown. ALT, alanine aminotransferase; AST, aspartate aminotransferase; ALP, alkaline phosphatase.

Parameters with significant difference between two groups were further analyzed using multivariate regression to conclude the independent risk factors for pneumonia development in pediatric patients. Resultantly, the length of hospital stay and the clinical symptom of coughing hold high odds ratio for further pneumonia acquirement. Additionally, the levels of AST (OR = 0.98, [95% CI,0.96–1.00], *p* = 0.034) and ALP (OR = 0.99, [95% CI,0.98–1.00], *p* = 0.043) upon hospital admission did not predict poor prognoses for pneumonia acquirement, given the fact that their OR values approached 1.00 (Table 6).

**Table 6:** Multivariable regression analysis of independent risk factors for pneumonia development in hospitalized pediatric patients.

	Odds ratio (OR)	95% Confidence interval (CI)	*p*
Basics			
Length of stay	1.36	1.12–1.67	0.002
Symptoms			
Cough	17.39	3.51–86.13	0.001
Expectoration	1.58	0.51–4.86	0.426
Co-infections			
Mycoplasma	2.74	0.23–33.31	0.429
Number of co-infections			
1	3.08	0.69–13.68	0.139
2	1.25	0.11–14.11	0.857
Blood cell count			
Lymphocytes, ×10^9^/L	1.37	0.96–1.97	0.087
Procalcitonin, ng/mL	0.45	0.06–3.14	0.417
AST, U/L	0.98	0.96–1.00	0.034
ALP, U/L	0.99	0.98–1.00	0.043
Sodium, mmol/L	1.14	0.96–1.36	0.135

**Figure 2:**
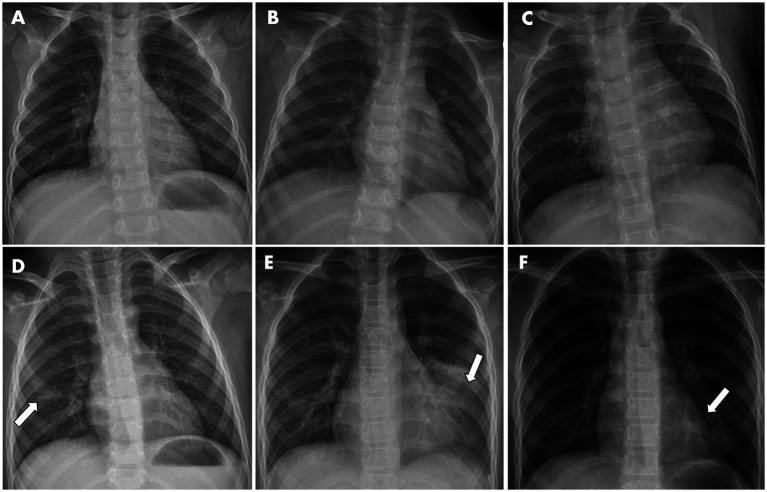
Representative X-ray graphs of pediatric inpatients infected with IAV, taken upon hospital admission, showed typical pathological changes in the lungs, without **(A–C)** or with **(D–F)** further pneumonia development. **(A)** From a 3-year-old girl with fever and cough symptoms. X-ray image showed the texture of both lungs was increased and slightly blurred. **(B)** From a 2-year-old girl having cough and fever. **(C)** From a 2-year-old boy with fever. Images showed the texture of both lungs was increased and slightly blurred, but no obvious exudative lesions were observed. **(D)** From a 6-year-old boy with fever. Image showed patchy faint shadow with blurred margins in the right lower lung region (indicated by arrow). **(E)** From a 9-year-old boy having cough and fever. Image showed a flocculent high-density shadow with blurred margins in the left middle and lower lung region (indicated by arrow). The morphology and size of the cardiac shadow were not abnormal, and the right rib-diaphragm angle was sharp with a smooth diaphragmatic surface. The left rib-diaphragm angle was shallow and obtuse. **(F)** From an 8-year-old girl with fever. Image showed a flocculent high-density shadow with blurred margins was observed in the left lower lung region (indicated by arrow), but no obvious exudation or solid changes in both lungs were noticed.

In parallel, available X-ray images upon hospital admission of 106 inpatients were collected and analyzed for radiological features. As a result, 5.7% images did not reveal noticeable abnormality in chest radiographs, while most inpatients exhibited the increased lung texture (94.3%) and to a lesser extent patchy shadow (40.6%). There was no statistical difference in X-ray patterns between the non-pneumonia and pneumonia groups upon hospitalization, albeit radiological features remain gold standards for pneumonia diagnosis. Representative images are depicted in Figure 2.

## Discussion

In this study we examined the independent risk factors that led to the hospitalization of pediatric patients by comparing the baseline characters of 798 outpatients and 208 inpatients infected with IAV. The sign of abdominal pain, the co-infection of IBV, and the abnormally high levels of lymphocytes and CRP, came into light as the independent risk factors of pediatric hospitalization due to IAV infection. We also retrospectively compared the clinical characteristics between the pneumonia and non-pneumonia groups to finalize the key determinants for pneumonia development among hospitalized pediatric patients. As a result, the length of hospital stay and the clinical symptom of coughing were attributed to be risk factors for pediatric inpatients to acquire pneumonia.

Influenza is an acute and contagious respiratory illness caused by the viral infection (16). Insofar, four types of influenza viruses that belong to the *Orthomyxoviridae* family have been identified (17). Among them, influenza A, B, and C viruses (i.e., IAV, IBV, and ICV) can circulate in humans, while influenza D virus only infects livestock animals (18). Being enveloped, negative-sense and single-strand RNA viruses, IAV and IBV are major viral burdens for the seasonal epidemics (18). In addition to annual epidemics, there have been four influenza pandemics recorded insofar, all caused by IAV subtypes (19). IAV contains genomic RNA segments that encode two vital proteins on the surface of viral particles; namely, haemagglutinin (HA) and neuraminidase (NA) (20). Genomic reassortment of these RNA segments will yield new IAV subtypes, initiating the pandemic and subsequently becoming the circulating strains (17). After the end of January 2023, H1N1 IAV dominates in most countries, including U.S. and China, followed by H3N2 (21–23). The median reproduction (*R*) number for seasonal influenza is estimated to be 1.28, whereas the median *R* value for 2009 H1N1 pandemic is 1.46 (24).

For individuals of all age, the infection rate of influenza is higher among children than adults (25). In China, the pediatric patients are responsible for the highest rates of influenza-related hospital visit and hospitalization (26). Our result here indicated that children of younger age were associated with higher risks of hospitalization, in agreement with others (27, 28). The female proportion was substantially higher in the pediatric inpatients than outpatients. This gender disparity in influenza patients was previously reported, as sex hormone may adjust the immune responses to viral invasion to a different degree (29). Higher female/male ratio was found with a link to higher hospitalization odds in children through puberty when infected by seasonal influenza, as well as poorer disease outcomes in adults of reproductive age infected by pandemic influenza (30).

Asymptomatic influenza infection is much less common in children than adults, because younger individuals are more likely to develop the relatively naïve immune response due to less viral exposure history (31). This was consistent with our result here as all inpatients and outpatients experienced typical signs of influenza, including fever, cough, sore throat, and stomachache, etc. (32). In addition, compared to the outpatients, the pediatric inpatients in our study showed symptoms of cough, vomiting, expectoration, and abdominal pain with much higher incidence, suggesting that virulence may disseminate into the lower respiratory tract, before it further impacts the gastrointestinal systems and other organs, either directly via blood stream or indirectly through immune cells (i.e., lymphocytes) (33, 34). Although most infections are self-limited in the patients’ upper respiratory tract, non-respiratory diagnoses have also been recognized, such as cardiological or neurological complications, and disease severity can range from asymptomatic, mild, moderate, critical, to even fatal (35). Seasonal influenza-caused hospitalization and death rate are high in children at the population level, and those rates generally decrease as their ages grow (36).

Hematological and biochemical abnormalities have been determined and used for diagnosis of influenza infection (37). Leukocytosis, neutrophilia, monocytosis, and lymphopenia, which were found in a substantial portion of patients here, have been frequently observed in pediatric and adult patients (5, 38, 39). Similar to our finding, elevated CRP level was associated with influenza infection in respirator tracts (40). This inflammatory biomarker, together with other biochemical indicators that signify the cardiac (e.g., CPK, LDH) and hepatic (e.g., AST, ALP) abnormality due to influenza infection, has been documented in the literature and recurred in this study (41, 42).

In our study, co-infection of IBV became common in the children infected with IAV, which also accounted for an important risk factor associated with pediatric influenza hospitalization. This finding stood in line with our previous report on adult influenza infections (20). Currently, influenza B/Victoria lineage that was predominant in China during prior winters, co-circulates with IAV subtypes (21, 43). The IBV circulation has been reported within the highest group of school-aged children (5–14 years old) (44, 45). Regardless of long-term co-circulation of IAV and IBV subtypes worldwide, co-infection of both viruses has been overlooked, especially for the consequent clinical severity among pediatric patients (46). In this study, dual infection rate of pediatric inpatients is much higher than that of outpatients, being evidence that IBV co-infection makes a significant contribution to the influenza burden in this period, which further lifts the risk of disease severity (47).

Pneumonia is an inflammatory pulmonary disease that could be infected by a variety of pathogens, including viruses, bacteria and fungi, while pediatric pneumonia remains a leading cause for childhood hospitalization and fatality worldwide (48). Attributes to acquiring pneumonia among hospitalized pediatric patients could be very diverse and complicated. Firstly, in this study the community-acquired pneumonia (CAP) could not be differentiated from the hospital-acquired pneumonia (HAP) that is initialized at least 48 h upon hospital admission. The exact scenario on which pneumonia is initially acquired greatly influences diagnostic and therapeutic approaches (49). Secondly, albeit all patients were IAV-infected, their individual viral-bacterial co-infection spectrum is unclear, further confounded by many unidentified factors such as pretreatment before hospital visit (e.g., using over-the-counter medications), vaccination type and history, and household conditions, etc. Those contribute to the difficulty of determining the risk factors for pneumonia development in hospitalized children. In this study we simply compared the baseline clinical characteristics between the pneumonia and non-pneumonia cohorts and further conducted multivariate regression analysis to decide the key determinants for pneumonia development; that is, the prolonged hospital stay that heightens the risks of nosocomial infection, and the clinical symptom of cough that signifies the tending infection in the low respiratory tract.

Still being the most cost-effective measure to prevent the influenza infection and severity, vaccination remains a low coverage in China’s population, mostly due to the lack of vaccine awareness or the concern of vaccine safety and effectiveness (50, 51). Only 11.9% of children aged 0.5–17 years received a recent influenza vaccine during 2015–2016 (52). The vaccination coverage among children under 5 years old was estimated to be <4% (53). On the other hand, in the 2010 influenza season, the post-licensure effectiveness of full influenza vaccination (two doses with at least 28 days apart) was reported to have 57.8% protection to children aged 0.5–5 years (54). Simultaneously, antibodies against influenza generated after natural infection or vaccine immunization decline over time, let alone for the antigenic drift of circulating viruses from season to season that can lead to reduced seroresponse (55). Moreover, the global influenza activity was low over the past three years amid COVID-19 pandemic, so the pre-existing immunity has significantly waned (56). This explains the soaring incidents of influenza infection after the COVID-19 restriction was lifted. Therefore, timely increase of influenza vaccine uptake, especially among the priority groups such as young children and older adults, is imperative.

Our study here has several limitations. First, all pediatric patients enrolled in this short duration were not tested for COVID-19 positivity, so the possibility of co-infection at the time cannot be excluded, since these two types of viral diseases share many similarities in common. The main purpose of this study is to analyze the clinical features of pediatric patients with seasonal influenza infection immediately after COVID-19 restriction was lifted. The choice of such a short period (from February to April 2023) is due to the flu-erupting season after three years’ COVID-19 measures ended in December 2022. Notably, the COVID-19 infection, if there is, may exacerbate the disease progression or interfere with the antiviral treatment, although the exact contribution remains unknown. Second, the information on influenza vaccination in this study is not available. Susceptible individuals with recent vaccination will lower their risks of influenza complication, which should have been taken into accounts. Third, for a simplicity, we eliminated many confounding factors, such as exclusion of cases where patients had serious underlying chronic diseases, such as the hematological diseases, and had compromised immune system. For the impact of influenza infection on those patient groups, questions regarding the risk factors associated with disease severity and mortality remain unsolved.

Despite of those limitations, the results from this retrospective study can be instructive in several ways. Firstly, different patient groups can be compared for multiple outcomes with different severities, including hospital admission and pneumonia acquirement. Secondly, the prognostic factors in association with pediatric hospitalization or pneumonia development could be very helpful to design and assess the future prospective studies on this aspect, to come up with better therapeutic strategies.

## Conclusion

We here reported the clinical characteristics of pediatric patients with IAV infection in China during February–April 2023, a flu-erupting season after 3 years’ COVID-19 measures ended. While younger age and higher female gender proportion were found in pediatric inpatients than outpatients, co-infection with IBV, symptom of abdominal pain, and certain hematological abnormality were determined to be independent risk factors for pediatric hospitalization. Furthermore, the cough symptom upon hospital admission and the length of hospitalization were the key determinants for pneumonia development.

## Data availability statement

The raw data supporting the conclusions of this article will be made available by the authors, without undue reservation.

## Ethics statement

The studies involving humans were approved by Research Ethics Committee of the Affiliated Hospital of Jiangsu University. The studies were conducted in accordance with the local legislation and institutional requirements. Written informed consent for participation in this study was provided by the participants’ legal guardians/next of kin.

## Author contributions

YZ: Data curation, Formal analysis, Investigation, Validation, Writing – original draft. XH: Data curation, Formal analysis, Investigation, Methodology, Software, Validation, Writing – original draft. JZ: Conceptualization, Formal analysis, Investigation, Methodology, Resources, Supervision, Writing – original draft, Writing – review & editing. ZT: Conceptualization, Formal analysis, Investigation, Methodology, Resources, Supervision, Writing – original draft, Writing – review & editing.
